# Novel Application of a Surgeon-Operated Clysis Delivery System in Burn Surgery

**DOI:** 10.3390/ebj3010020

**Published:** 2022-03-21

**Authors:** Alexander Morzycki, Peter O. Kwan, Edward E. Tredget, Joshua N. Wong

**Affiliations:** Division of Plastic and Reconstructive Surgery, University of Alberta, Edmonton, AB T6G 2R3, Canada; morzycki@ualberta.ca (A.M.); peter.kwan@ualberta.ca (P.O.K.); etredget@ualberta.ca (E.E.T.)

**Keywords:** clysis, intra-operative hemodynamics, transfusion sparing, cost-analysis

## Abstract

Insufflation of epinephrine-containing solutions (clysis) has shown to decrease blood loss in burn surgery. Current delivery methods are associated with significant cost and may predispose burn patients to hypothermia. This was a proof-of-concept study to evaluate a novel surgeon-operated clysis delivery system. Our initial experience with a novel fluid management system is presented. Temperature, pressure, and volume of clysis was recorded. Patient and burn factors were evaluated and complications collected. Finally, a cost-effectiveness analysis was conducted. Thirty-seven consecutive cases comprising 22 adult patients (15/22, 68% male), with a mean age of 49 years (+/−19) were reviewed. The mean % total body surface area of all patients was 39 (+/−21.7). The mean temperature, pressure, and volume of administered clysis was 32.2 degrees Celsius (+/−4.4), 265.04 mmHg (+/−56.17), and 5805.8 mL (+/−4844.4), respectively. The mean dose of epinephrine administered was 14.5 mg (+/−12.1). The mean temperature variability was 1.1 °C (+/−1.2). The total mean of packed red blood cells (PRBC) transfused was 507.6 mL (+/−624.4). There were no recorded complications. We identified a cost savings of CAD 20,766 over the cases examined, compared to our conventional clysis delivery technique. This novel technique provides rapid and safe infiltration of warmed clysis in burn surgery. We were able to maintain intra-operative euthermia. In addition, this technique may be transfusion-sparing. The introduction of this method of clysis administration was associated with significant cost-savings. Future randomized study is necessary.

## 1. Introduction

Tangential excision of major burns is associated with significant blood loss [1,2,3,4]. Various techniques and agents to prevent intra-operative blood loss have been employed and studied that include the use of tourniquets, local anesthetics, thrombin, fibrin sealants, systematic administration of blood products, use of electrocautery, and infiltration of vasoactive solutions (clysis) [5,6].

Clysis of epinephrine-containing solutions for autograph harvest and burn excision have been shown to be safe and effective [7,8]. Reported benefits include decreased intra-operative blood loss, improved post-operative pain control, and a taut, level surface for excision and skin graft harvest [7,8,9]. Historically, intra-operative clysis has been delivered manually using “Pitkin” syringes or a pneumatic tourniquet method [10,11]. These methods have significant disadvantages including slow rate of infiltration, operator fatigue, first basilar joint arthritis, and poor control of the temperature of infiltrate. In addition to evaporative water loss and suppression of innate reflexes to conserve body temperature intra-operatively due to general anesthesia, infiltration of cold clysis can further contribute to the morbidity and mortality associated with hypothermia in burn surgery. The maintenance of euthermia in burn surgery that uses clysis is therefore paramount.

The purpose this proof-of-concept study was to describe the novel application of a surgeon-operated clysis delivery system (Thermedx) (Thermedx LLC, Cleveland, OH, USA) in burn surgery, and its effect on intra-operative thermoregulation and hemostasis. Additionally, we present a cost analysis of this new method.

## 2. Materials and Methods

We conducted a retrospective review of all consecutive major adult (>18 years of age) burn operations performed over a one-year period (August 2019–August 2020) at a tertiary American Burn Association accredited burn center. Only cases applying the Thermedx system were included. We collected perfusion-related (temperature, pressure, volume of clysis, and amount of epinephrine administered) and patient-related (% total body surface area burn (%TBSA), sex, age, body mass index (BMI), duration of surgery, intra-operative temperature, and administration of intra-operative blood products) characteristics. Adverse events were reviewed as well.

A cost-analysis was performed from a third-party payer perspective comparing the use of a Thermedx system to a traditional perfusionist-operated roller pump method. The mean duration of surgery for the index cases presented were first calculated. The theoretical cost of utilizing the perfusionist-operated roller pump system was then calculated based on these figures.

Written patient consent was obtained for the case presented.

### 2.1. Statistical Analysis

Multiple linear regression was performed to determine the effect of %TBSA, age, BMI, surgery duration, and temperature of clysis, on intra-operative core body temperature. We also examined the effect of %TBSA, BMI, surgery duration, age, temperature, and volume of clysis administered, on intra-operative blood product requirements. All analyses were conducted using SPSS version 26.0 (IBM, Armonk, NY, USA).

### 2.2. Technique 

**Figure 1:**
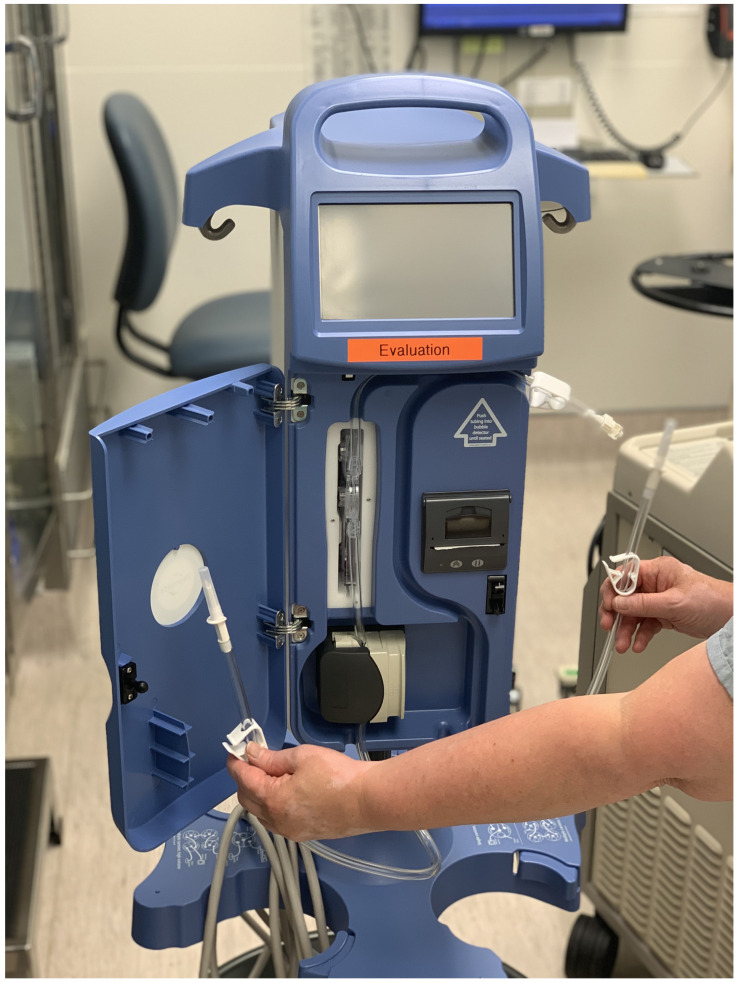
Thermedx fluid management system.

All patients were brought to the operating theater and placed under general anesthesia. The senior burn surgeon was responsible for determining the % TBSA and extent of burn to be excised. A solution containing 7.5 mL epinephrine 1:1000 per 3L of normal saline (NS) (2.5 mg/mL epinephrine) was then infiltrated beneath the burn eschar in the subdermal plane using a Thermedx Fluid Smart Fluid Management System (Thermedx LLC, Cleveland, OH, USA) (Supplementary Video S1. Technique video of the Thermedx fluid management system). The solution was selected as this is an institutional standard but can be altered to fit the surgeon’s preference. We used a concentration of 1:400,000 epinephrine to retain maximal vasoconstrictive benefits. The system is connected to multi-perfuser high flow extension tubing and four separate spinal needles (Figure 1). Taut burn eschar requiring excision was the end point of insufflation. Fluids were warmed to 32 °C prior to commencing insufflation. Fluid temperatures were not titrated to %TBSA or current body temperature.

Burn excisions were performed in a sequential tangential manner using a mixture of electrocautery, Braithwaite, and/or Weck/Goulian blade, depending on the depth and region of burn. Once adequate hemostasis was achieved, immediate autografting was performed.

Additional clysis was then infiltrated beneath the region to be harvested for autografting. The endpoint of infiltration was uniform blanching over the donor sites. The endpoint of infiltration was determined by the senior surgeon.

## 3. Results

Thirty-seven consecutive cases comprising of 22 adult patients (15/22, 68% male), with a mean age of 49 years (+/−19), met inclusion criteria. The mean % TBSA of all patients was 39 +/− 21.7. Weight and BMI of included patients were 75.7 kg (+/−18.4) and 25.2 (+/−6.5), respectively. Surgery duration ranged from 120 to 675 min (mean 331.1 +/− 124.6). The mean temperature, pressure, and volume of administered clysis was 32.2 °C (+/−4.4), 265.04 mmHg (+/−56.17), and 5805.8 mL (+/−4844.4), respectively. The mean dose of epinephrine administered was 14.5 mg (+/−12.1).

The mean temperature variability was 1.1 °C (+/−1.2). The total mean of packed red blood cells (PRBC) transfused was 507.6 mL (+/−624.4), or 2.0 units +/−2.50. A single transfusion of 996 mL of fresh frozen plasma was administered to one patient.

We did not find any effect of BMI, duration of surgery, and temperature of administered clysis solution on intra-operative decrease in basal temperature. Higher %TBSA was associated with greater degree of intra-operative temperature variability (95% CI: 0.00–0.044, *p* < 0.05).

Patients with higher %TBSA (95% CI: 3.16–23.60, *p* < 0.05) were more likely to require intraoperative transfusions. There was no effect of BMI, age, volume and temperature of administered clysis, or length of surgery on the likelihood of requiring intra-operative transfusion.

No adverse reactions such as cardiac dysrhythmias, adrenergic-associated events, compartment syndrome, or inadvertent administration of clysis into the thoracic or abdominal cavity with the Thermedx system were identified.

### Cost Analysis

We performed a cost analysis based on a third party-payer system (Table 1). The use of the Thermedx clysis delivery system was associated with a cost savings of CAD 561.24 per case, based on the mean calculated operative time in this series. Over the course of the year, this amounted to a cost savings of CAD 20,766.

## 4. Discussion

Early excision and grafting of major burns are associated with significant bleeding [2,3,5,12,13]. Poor peri-operative thermoregulatory status may exacerbate blood loss and the need for blood product transfusions [14], potentially leading to poor outcomes associated with over-transfusion [15]. Numerous techniques to limit operative blood loss in burn surgery have been studied. Among these, the administration of vasoconstrictor clysis has been shown to be effective in burn excision [6] and skin graft harvest [11,16,17].

This a proof-of-concept study demonstrating a novel, safe, and cost-effective technique for the administration of vasoconstrictor clysis in major burn surgery. Using the Thermedx system, warmed and epinephrine-containing solution can be administered at a rapid rate with consistent temperature and pressure. Unlike with traditional bolus instillation, this novel method circumvents the need for frequent fluid bag replacement, manual injection, or variable pressure monitoring. Furthermore, multiple infusion lines allow for rapid instillation to numerous sites at one time, potentially decreasing operative time.

While a number of studies have described the effectiveness of vasoconstrictor clysis administration, including its transfusion-sparing properties in major burn surgery [4,11,18,19,20], we are only aware of one other study examining its effect on intra-operative temperature [21]. This study, however, is limited significantly by sample size (eight adult patients). In our study, clysis was administered at constant euthermic temperatures (32.2 +/− 4.4 °C). This allowed for a significant reduction in intra-operative temperature variability (1.1 +/− 1.2 °C), and prevention of intraoperative hypothermia, with mean intra-operative temperature lows of 36.20 +/− 1.02 °C. In a recent study of 1111 patients, Ziolkowski et al. (2017) showed that patients who experienced peri-operative hypothermia (<35 °C) were more likely to develop sepsis, pneumonia, urinary tract infections, and wound infections than those who maintained euthermia. This same cohort of patients who experienced hypothermia were at a higher risk of developing acute respiratory distress syndrome, deep vein thrombosis, and were at a higher risk of death [22]. The administration of warmed clysis may not only prevent intra-operative hypothermic episodes, but contribute to active patient warming, preventing the need for more invasive techniques [23].

Total blood loss resulting from tangential excision of burns is difficult to estimate [24]. While this study does not examine the estimated blood loss (EBL) during surgery, we examined the administration of intra-operative blood products as a surrogate measure. In a retrospective study of blood bank usage in burn surgery, Yogore et al. (2006) showed that patients with higher %TBSA burns received more blood product transfusion, and per procedure, patients received on average of 3.3 +/− 3.5 units of PRBC. While these results are consistent with our findings showing higher transfusion volumes among patients with higher %TBSA burns, patients only required, on average, 2.0 units +/− 2.50 per procedure. Although these results demonstrate promising vasoconstrictive properties of clysis on blood loss, our study ultimately lacks a comparison arm. Future, prospective investigations are needed to compare this novel technique to the standard of care.

A potential untoward consequence of utilizing the Thermedx system is the high volume of insufflated epinephrine. While isolated reports have shown morbidity at doses as low as 4 mg, [25] in this series, the mean volume of epinephrine safely administered was 14.5 mg. We reported no adverse adrenergic-related events and no cardiac disrythmias. While maximum doses of subcutaneous epinephrine have been reported as 7–8 mg, we suspect that large volumes of instilled clysis are removed rapidly during burn excision, prior to systemic absorption. This may allow for permissive over-insufflation of tissues. In addition, the vasoconstrictive effect of the epinephrine may delay its systemic redistribution from the sub-dermal space much like tumescence in lipectomy procedures. It should be noted, however, that inadvertent intra-thoracic, intra-abdominal, or intra-compartmental infiltration may lead to serious consequences. Though none of these adverse events occurred in this study, careful positioning of the spinal needle for insufflation of clysis is essential.

The treatment of burns is associated with significant cost [26,27]. In an era of fiscal prudence and responsibility, the search for cost-saving strategies is necessary. With the recent introduction of the Thermedx clysis administration system, we were able to report a cost savings of CAD 20,766 in the index year, as compared to the cardiac bypass roller pump system, which was historically used at our institution. While the system is associated with a significant upfront investment, it has a low cost-per-case ratio. Additionally, the system has multiple applications in urological and gynecological surgery, allowing us to share this technology and its upfront costs with colleagues in multiple subspecialty departments. Unfortunately, our cost-analysis has shortcomings. Costs are only determined based on mean operative time utilizing the novel Thermedx system and lack a matched cohort. Furthermore, we do not have any patient specific outcome data to include in our model, limiting our ability to perform a cost-effectiveness or cost-utility analysis. We hope to address these concerns in a subsequent comparative analysis.

In addition to the limitations outlined above, our study did not collect any specific burn-outcome data. [6] Ultimately, further longitudinal research is necessary to examine the association between administered clysis on graft survival and other important patient-specific outcomes.

## 5. Conclusions

By using the Thermedx system in the delivery of clysis in burn surgery, we were able to achieve effective hemostasis and maintain core temperature control in patients with moderate to large burns. This novel technique was associated with significant cost-savings and may be an economically feasible addition at other centers caring for burn patients. Future randomized comparisons are ultimately required to confirm the results of this preliminary study.

## Figures and Tables

**Table 1:** Cost-analysis.

Breakdown of Costs	Equipment	Thermedx ^®^	Cardiac Bypass System
Equipment	Tubing	CAD 145.00	CAD 407.16
	Multi-perfusor	CAD 18.90	
	Hi-flow extension	CAD 32.44	
	Spinal needles	CAD 8.16	
Perfusionist			CAD 65.00/h
Total cost (per case)		CAD 204.50	CAD 765.74
Cost difference		–561.24 CAD	
Cost savings (Cases examined)		20,766 CAD	

CAD: Canadian dollar; Thermedx: Thermedx LLC, Cleveland, OH, USA.

## Data Availability

Data is available upon request.

## Supplementary Materials

The following supporting information can be downloaded at: https://www.mdpi.com/article/10.3390/ebj3010020/s1, Video S1. Technique video of the Thermedx fluid management system.
